# Small bowel perforation due to indistinguishable metastasis of angiosarcoma: case report and brief literature review

**DOI:** 10.1186/s40792-016-0169-y

**Published:** 2016-05-08

**Authors:** Tomoyuki Uchihara, Yu Imamura, Shiro Iwagami, Ikko Kajihara, Hisashi Kanemaru, Ryuichi Karashima, Satoshi Ida, Takatsugu Ishimoto, Yoshifumi Baba, Yasuo Sakamoto, Yuji Miyamoto, Naoya Yoshida, Masayuki Watanabe, Ken-ichi Iyama, Hironobu Ihn, Hideo Baba

**Affiliations:** Department of Gastroenterological Surgery, Kumamoto University Graduate School of Medical Sciences, 1-1-1 Honjo, Chuo-ku, Kumamoto, 860-8556 Japan; Department of Dermatology and Plastic Surgery, Faculty of Life Sciences, Kumamoto University, 1-1-1 Honjo, Chuo-ku, Kumamoto, 860-8556 Japan; Department of Gastroenterological Surgery, Cancer Institute Hospital of Japanese Foundation for Cancer Research, 3-8-31, Ariake, Koto, Tokyo, 135-8550 Japan; Department of Surgical Pathology, Kumamoto University Hospital, 1-1-1 Honjo, Chuo-ku, Kumamoto, 860-8556 Japan

**Keywords:** Angiosarcoma, Intestinal metastasis, Intestinal perforation, Oncologic emergency

## Abstract

Intestinal metastasis of angiosarcoma is extremely rare. We herein report a case of intestinal perforation due to intestinal metastasis of angiosarcoma. The patient was a 72-year-old Japanese man with multiple recurrent angiosarcomas of the scalp. He developed acute abdominal pain with guarding, and we performed an emergency exploratory laparotomy. An intestinal perforation was found 80 cm from the ligament of Treitz, and partial jejunectomy was successfully performed. Macroscopic inspection revealed no obvious injury, ulcer, or tumor at or around the perforation site. Pathological examination revealed angiosarcoma cells penetrating through all layers of the jejunum at the site of intestinal perforation. This is the first reported case of intestinal perforation caused by indistinguishable intestinal metastasis of angiosarcoma. This case emphasizes intestinal metastasis of angiosarcoma as a possible cause of small bowel perforation in patients with advanced angiosarcoma, even when no visible tumor is present during surgery.

## Background

Angiosarcoma is a very rare tumor that originates from endothelial cells and constitutes approximately 2 % of soft tissue sarcomas [1]. Angiosarcoma most commonly occurs in the cutaneous tissues of the head, neck, and face, particularly the scalp [2]. The prognosis of this tumor is poor [1]. The most common sites of recurrence are the regional lymph nodes and lungs, followed by the liver and spleen [3].

The incidence of malignant tumors has been increasing worldwide, and the management of abdominal oncologic emergencies has thus become more clinically important. Common causes of abdominal oncologic emergencies are obstruction, hemorrhage, and perforation. Among these, intestinal perforation often requires urgent surgical intervention. Lung and gynecologic cancers occasionally cause intestinal perforation by metastatic tumors [4, 5].

We herein report an extremely rare case of intestinal perforation due to intestinal metastasis of angiosarcoma, which was indistinguishable during the operation but pathologically confirmed after surgery. We also provide a brief review of the literature regarding intestinal metastasis of angiosarcoma.

## Case presentation

**Fig. 1:**
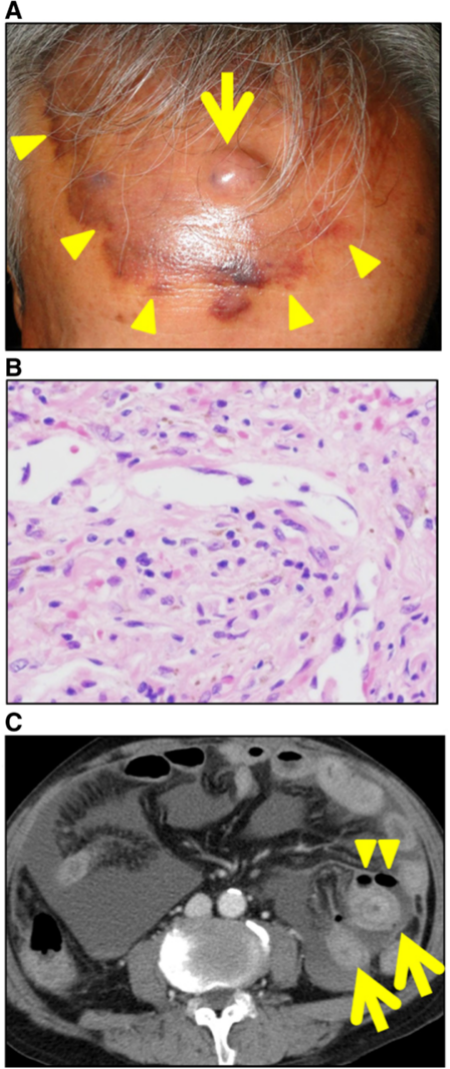
Preoperative findings. **a** The primary site of angiosarcoma of the scalp shows infiltrative spread. An *arrow* shows the nodule. The tumor is demarcated by the *red-brown color* (*arrowheads*). **b** Tumor cells from the primary site of the scalp (biopsy specimen). **c** Preoperative findings on contrast-enhanced computed tomography. Focal wall thickening of the small intestine is surrounded by ascites (*arrows*), and free air is present (*arrowheads*)

A 72-year-old Japanese man presented with recurrent angiosarcoma. The primary tumor was located on the scalp and exhibited infiltrative spread with a single elevated nodule (Fig. 1a, b). He had metastases of the liver (multiple), lymph nodes (along the left spinal accessory nerve), bone (fourth thoracic vertebra), and muscle (left latissimus dorsi). Although the disease was stable with the first line chemotherapy of triweekly paclitaxel, it was discontinued due to a severe peripheral neuropathy. After the initial course of the second-line chemotherapy using docetaxel, he exhibited acute abdominal pain. On physical examination, the patient was pale, diaphoretic, and in distress due to continuous abdominal pain. He had a temperature of 36.6 °C, heart rate of 93/min, and blood pressure of 97/61 mmHg. Significant findings included pale conjunctiva and hypogastrium tenderness with guarding. Laboratory examination showed a hemoglobin level of 7.2 g/dL, white blood cell count of 10,100/μL, platelet count of 587 × 10^3^/μL, and C-reactive protein level of 16.64 mg/dL. Contrast-enhanced computed tomography showed focal wall thickening of the small intestine surrounded by ascites and small collections of free air (Fig. 1c). These findings indicated that the peritonitis had been caused by intestinal perforation.

**Fig. 2:**
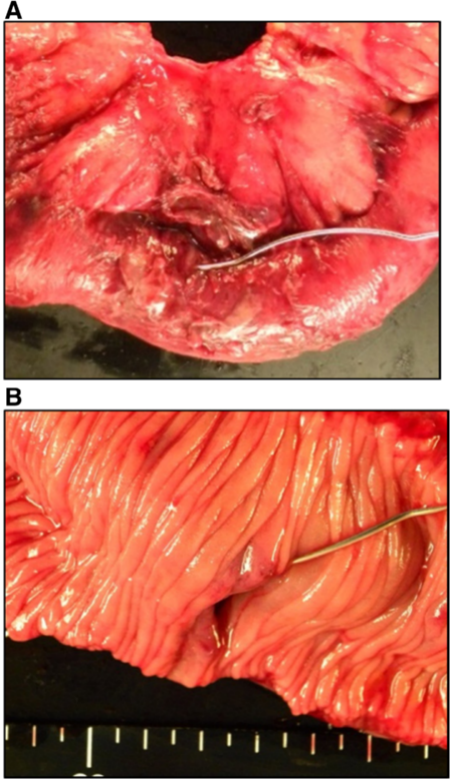
Macroscopic findings of resected small intestine. **a** No obvious injury or tumor is present on the serosal surface at or around the perforation site. **b** No obvious ulcer or tumor is present on the mucosal surface at or around the perforation site

We performed an emergency exploratory laparotomy. A large amount of bloody ascites was observed, although there were no visible or palpable metastatic tumors in the peritoneal cavity. Jejunal perforation was found 80 cm from the ligament of Treitz. Intraoperative examination revealed no obvious injury, ulcer, or tumor at around the perforation site (Fig. 2a, b). A 50-cm-long segment of jejunum, including the perforation site, was surgically removed. End-to-end anastomosis was then performed by a hand-sewing technique. The patient began oral ingestion on postoperative day 4, and his postoperative course was favorable until postoperative day 8. Hemothorax was suddenly developed on postoperative day 9. It was caused by pleural metastasis of angiosarcoma, because pleural effusion was positive for angiosarcoma cell. Further surgical intervention was not considered because of the patient’s unstable condition due to multiple organ metastases. On postoperative day 23, he died of hemorrhagic shock due to hemothorax.

**Fig. 3:**
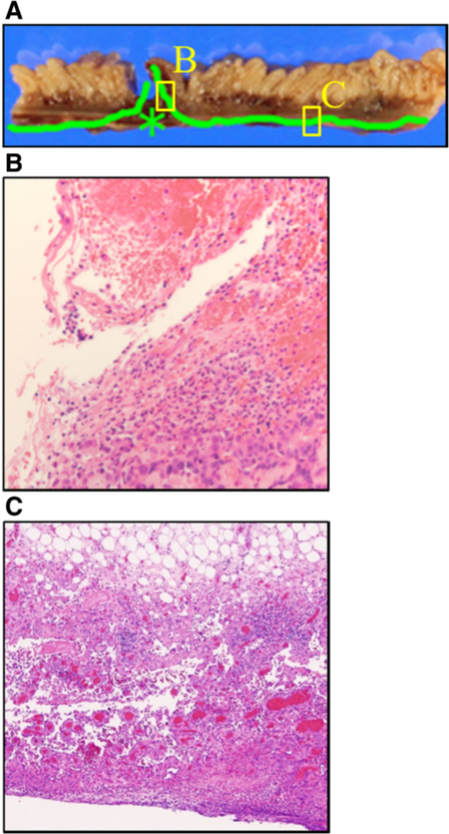
Pathological findings of resected small intestine. **a** Macroscopic cross section and tumor cell mapping (*green*). An *asterisk* shows the site of perforation. **c** Tumor cells are invading through all layers of the jejunum with necrotic change. **c** Tumor cells are horizontally infiltrating the subserosal layer of the jejunum around the perforation site

Pathological examination revealed that the intestinal perforation was due to a metastatic tumor of angiosarcoma. On microscopic examination, angiosarcoma cells were invading through all layers of the jejunum at the perforation site, which was surrounded by necrotic changes (Fig. 3a, b). Interestingly, tumor cells were horizontally infiltrating within the subserosal layer of the jejunum around the perforation site (Fig. 3c). The tumor cells in the metastatic region (Fig. 3c) were identical to those at the primary site (Fig. 1c).

### Discussion

To date, only six cases of surgical resection of intestinal metastasis of angiosarcoma have been reported (Table 1) [3, 6–10]. To the best of our knowledge, ours is the first case of intestinal perforation due to macroscopically indistinguishable metastasis of angiosarcoma (Table 1).

**Table 1:** Review of reported seven cases (including this case) with intestinal metastasis of angiosarcoma

Authors, year of publication (reference number)	Age, sex	Primary tumor site	Site of intestinal metastasis	Symptom at presentation	Visible or tactile tumor at the site of perforation	Operative procedure	Outcome and time after surgery
Schmid E et al., 1984 [6]	75, M	Aorta, bone	Terminal ileum	Hemorrhage	Present (visible hemorrhage)	Ileocecal resection	Dead, approximately 14 months
Kunkel D et al., 1993 [7]	―	Aorta	Massive small intestine	Hemorrhage	―	―	―
Bandorski D et al., 2002 [9]	75, M	Thyroid	Massive small intestine	Hemorrhage	―	Multiple jejunal and ileal resection	―
Hsu JT et al., 2005 [10]	49, M	Spleen	Small intestine	Hemorrhage	Present (visible hemorrhage)	Partial intestinal resection	Dead, 7 months
Ruffolo C et al., 2004 [3]	67, M	Scalp	Small intestine	Perforation (due to tumor ulceration)	Present (tactile ulcer)	Intestinal wedge resection	Dead, 16 days (due to respiratory distress)
Santonja C et al., 2001 [8]	64, F	Aorta	Ileum	Perforation (due to tumor cell embolization in intestinal artery)	Absent	Right ileocolectomy	Dead, 18 days (due to acute renal failure, and liver infarction)
Uchihara et al., 2015 (Current case)[22]	72, M	Scalp	Jejunum	Perforation (due to invisible metastatic-tumor-cell penetration)	Absent	Partial jejunal resection	Dead, 23 days (due to hemorrhagic shock due to hemothorax)

“―” means no data available

According to our literature review, hemorrhage is a common initial symptom in patients with intestinal metastasis of angiosarcoma. Hemorrhage occurred in four cases (67 %), but peritonitis occurred in only two cases (33 %) (Table 1). Ruffolo et al. [3] reported a case of intestinal perforation due to invasion of an ulcerated tumor through all layers of the small intestine. In the present case, no visible or palpable ulcerative changes were present on the mucosal or serosal surface of the intestine at or around the perforation site. Only the pathological examination revealed intestinal metastasis of angiosarcoma. Santonja et al. [8] also reported a rare case of intestinal perforation caused by intestinal ischemia due to tumor cell embolization, but not due to direct intestinal metastasis. In their case, the primary tumor was located in the abdominal aorta, and the tumor cells spread into small mesenteric arteries, resulting in intestinal infarction. In the present case, there was no evidence of tumor cell embolization on pathological examination.

The growth pattern of angiosarcoma is usually infiltrative, without the formation of a capsule or clear border distinguishing the tumor from normal tissue. At the primary site (scalp) in the present case, a red-brown color was the only clue of demarcation between the tumor and normal tissue (Fig. 1a). Such a subtle color change may be more difficult to detect in a perforated intestine with inflammation than in the skin. In fact, although many tumor cells were present within the serosal layer at or around the perforation site, we found no metastatic changes in the intestine (Fig. 2a, b). Pathological examination would be necessary to confirm the presence or absence of metastatic angiosarcoma of the intestine.

Intestinal perforation during chemotherapy can be explained by necrotizing enteritis in the presence of neutropenia, metastatic tumor infiltration, and direct intestinal damage by chemotherapeutic agents characterized by mitotic arrest [11–14]. In the present case, there was no evidence of neutropenia, enteritis, or any histological findings of damaged cells with mitotic arrest. Although we cannot exclude the possibility of tumor necrosis by chemotherapy, the intestinal perforation in this case may be due to a metastatic tumor invading the whole wall of the intestine.

The prognosis of angiosarcoma is very poor. Lahat et al. reported a median disease-free survival duration of 43 months (range, 1–188 months), a 5-year disease-specific survival rate of 35 to 40 %, and a median survival duration of 10 months in patients with metastatic angiosarcoma [1, 15–18]. The prognostic factors of angiosarcoma are reportedly a large tumor (>5 cm) [19, 20], old age, distant metastasis, and poor performance status [19, 21]. In our review of seven cases (six previously reported cases plus ours) of intestinal metastasis from angiosarcoma, the prognosis of patients with peritonitis was remarkably poorer than that of patients with hemorrhage alone. Additional evidence is necessary to establish the surgical indications for intestinal perforation due to metastatic angiosarcoma.

## Conclusions

In summary, intestinal perforation due to intestinal metastasis of angiosarcoma should be taken into account, in abdominal emergency cases with advanced angiosarcoma.

## Consent

When obtaining an informed consent for surgical procedure, a general consent was also obtained from the patient, for publication and presentation, as usual.
